# Seasonal changes of rumen and intestine morphology of the Qinghai yak (*Bos grunniens*)

**DOI:** 10.14202/vetworld.2018.1135-1138

**Published:** 2018-08-16

**Authors:** Bao A. Ding, Shuang Q. Ma, Zong R. Li, Xi L. Li, Stephen R. Madigosky

**Affiliations:** 1 State Key Laboratory of Plateau Ecology and Agriculture, College of Agriculture and Animal Husbandry, Qinghai University, Xining 810016, China; 2 Department of Environmental Science and Biology, One University Place, Chester, PA, 19013, Widener University. USA

**Keywords:** green grass, intestine, morphology, Qinghai yak, rumen

## Abstract

**Aim:**

The aim of the current study was to investigate the effects of seasonal changes in grass quality on the ruminal and intestinal morphology of male Qinghai yaks.

**Materials and Methods:**

A total of four male yaks with the same age of 4 years old from each season (summer and winter) were randomly selected and slaughtered to determine the effect of different season on intestinal morphology of yak in the Qinghai-Tibetan Plateau.

**Results:**

The histological analysis shows that male yak has the longer and wider papillae in rumen in green season. The height of villi in duodenum and jejunum was significantly higher in green season, and the width of villi on duodenum, jejunum, ileum, and rectum was significantly wider in green season. Surface area of villi and crypt depth in duodenum, jejunum, and ileum was significantly larger and deeper in green season. Submucosa thickness of duodenum, jejunum, ileum, and rectum was significantly thicker in green season. The muscular thickness of jejunum, cecum, and rectum was significantly thicker in green season.

**Conclusion:**

According to this research, we found that the seasonal changes of ruminal and intestinal morphology of yak showed different length and width papillae, villi, crypt, and submucosa. This fact was confirmed the functional advantages resulting from the ability to successfully adapt to a dry climate and diets, flat, open, and cold grassland may allow yak to overcome both water shortage and energy deficiency in winter.

## Introduction

A unique species resource has been formed in Qinghai-Tibetan Plateau, China, under unique climatic conditions and geographical environment. In the Qinghai-Tibetan Plateau animals, face cold weather, low oxygen, short grass period, high radiation, and harsh condition. Plateau species showed strong resistance characteristics. The yak (*Bos grunniens*), as a year-round grazing animal, is one of the unique livestock in the world. Yak physiology is well adapted to high altitudes, having larger lungs and heart than cattle found at lower altitudes, as well as greater capacity for transporting oxygen through their blood [1] due to the persistence of hemoglobin throughout life [2]. Conversely, yaks do not thrive at lower altitudes [3] and begin to suffer from heat exhaustion above about 15°C (59°F). Further, adaptations to the cold include a thick layer of subcutaneous fat and an almost complete lack of functional sweat glands [1].

Gastrointestinal tissues are critical components of the post-absorptive system as they mediate absorption of nutrients and play a role in the regulation of metabolite availability to all other tissues in the body. The ruminal and intestinal tissue can be different and therefore affect the inflow of absorbed nutrients into intermediary organs. In intensively reared cattle receiving higher amounts of concentrates not only the absorption surface of rumen papillae but also the height of duodenal and jejunal villi were seen to increase. This fact was confirmed by the positive correlation between the morphometric parameters of ruminal and intestinal mucosa [4]. In cattle, it has been demonstrated that the starch level in diet did not significantly affect small intestine morphology, but it affected rumen morphology [5].

Moreover, differences in villi and crypts morphology among several breeds cattle were reported [6]. The yaks were grazed on the natural grassland. The various rangelands of the plateau are characterized by their high altitude, short growing season (from June to September), and great seasonal variation in feed supply [7,8].

Although many studies showed that the physiology of the yaks corresponded with that of other domestic animals, whereas its body shape was different [8-11], less is known about various seasonal modulation patterns in morphological differences in digestive tract of yak between dry season and green season.

Thus, the objective of this study was to investigate the morphology change of the yak rumen and intestine during the dry season and green season in the Qinghai-Tibetan Plateau.

## Materials and Methods

### Ethical approval

The samples were removed for routine research scopes, following a procedure approved by the Ethics Committee of the University of Qinghai

### Animals and sample collection

Yaks were grazed on grassland in Henan county, Huangnan Tibetan Autonomous Prefecture, Qinghai Province, China. There are two seasons in terms of the situation of grassland. During summer (from June to September, average temperature 9.6°C), the animals of the reared group grazed on a summer pasture (with plenty of green grass and water), but from later autumn to next early spring (in particular, from the end of October to the middle of May, average temperature −1.8°C), they were grazed in a winter pasture (with dry hay and snow). Most of plants in Qinghai pasture are *Kobresia myosuroides* and *Elymus nutans*. Supplement diet at night was composed of wheat bran 50%, rapeseed dregs 10%, and hay 40%. Four male yaks with the same age (4 years old, good health status) from each season (4×2=8) were randomly selected and slaughtered to determine the effect of different season on intestinal morphology of yak in the Qinghai-Tibetan Plateau. Farmer uses female animals for production so we used male animals only in the study. Samples were taken on the first of March and the first of December.

### Histological analysis of the intestinal morphology

Rumen and segments of approximately 5 cm of duodenum, jejunum, ileum, cecum, and rectum were obtained before the removal of the entire rumen and intestinal tract. Samples were fixed in 4% buffered formalin solution for 24 h and processed for routine paraffin embedding. Serial 4 µm transverse sections were cut and stained with hematoxylin and eosin for morphological evaluation. Sections were examined using a light microscope (Leitz, Diaplan) connected to a PC through a Nikon digital system (Digital Sight DS-U1). Images were acquired using the NIS-Elements F version 2.10 software (Nikon) per transverse section of rumen and small intestine. Measurements were made using Image J 1.37V software (National Institutes of Health). 10 well-oriented and intact crypt-villus units of each slide were measured in triplicate. The villi height was defined as the distance from villus tip to crypt junction. The crypt depth was defined as the depth of the invagination between adjacent villi. The muscle thickness was measured from the junction between the submucosal and muscular layers to that between the muscular layer and the tunica serosa. The thickness of intestinal wall was defined as the distance from intestinal external subserosa to the muscular junction layer and submucosa.

### Statistical analysis

Mean values generated from all individual data were statistically analyzed by a one-factor variance analysis using the GLM procedure of the SAS Institute (Cary, NC, USA). Results are expressed as the mean ± standard error. If the main effect between any group was found to be significant, they were then compared using the Tukey’s multiple range test at p<0.05.

## Results

### The number, length, and width of papillae and muscular thickness of rumen of yak in different grazing seasons

The number, length, and width of rumen papillae during dry season and green season are summarized in Table-1. Although the number of rumen papillae and muscular thickness of rumen did not significantly change between dry season and green season (p>0.05), the length and width of rumen papillae significantly increase in green season compared to that in dry season (p<0.05).

**Table-1:** The number, length, and width of papillae and muscular thickness of rumen of yak in different grazing seasons.

Parameters	Dry season	Green season	p-value
The number of papillae (n/cm^2^)	82.59±2.75^A^	78.37±1.22^A^	0.168
Length (mm)	5.54±0.21^B^	11.82±0.36^A^	0.002
Width (mm)	1.32±0.02^B^	2.78±0.08^A^	0.034
Muscular thickness	379.91±14.91^A^	386.72±16.68^A^	0.284

Means within rows with different superscript capital letters at same day are different (p<0.05)

### The height of villi of the yak intestine in different grazing seasons

The height and width of villi during dry season and green season are summarized in Table-2. The height of villi in duodenum and jejunum was significantly higher in green season than that in dry season (p<0.05); however, the height of villi in ileum, cecum, and rectum was not different (p>0.05). The width of villi on duodenum, jejunum, ileum, and rectum was significantly greater in green season than it in dry season (p<0.05), on the contrary, in the cecum was lower in green season than in dry season (p<0.05).

**Table-2:** The height of villi (µm) in the intestine of yak in different grazing seasons.

Sections	Height of villi		p-value	Width of villi		p-value
	
	Dry season	Green season		Dry season	Green season	
Duodenum	518.47±23.39^B^	791.17±38.28^A^	0.012	49.64±1.83^B^	79.295±2.67^A^	0.009
Jejunum	1246.20±52.53^B^	1482.78±71.70^A^	0.043	142.78±6.54^B^	273.291±12.03^A^	0.028
Ileum	702.77±35.30^A^	696.38±22.54^A^	0.368	98.24±4.36^B^	145.709±6.25^A^	0.011
Cecum	718.18±33.25^A^	729.45±42.68^A^	0.714	101.51±4.16^A^	76.36±2.78^B^	0.046
Rectum	578.05±26.89^A^	523.24±36.28^A^	0.502	63.56±1.95^B^	283.684±10.41^A^	0.033

Means within rows with different superscript capital letters at same day are different (p<0.05)

### The surface area of villi and crypt depth in the intestine of yak in different grazing seasons

The surface area of villi and crypt depth is summarized in Table-3. The surface area of villi in duodenum, jejunum, and ileum was significantly larger in green season than that in dry season (p<0.05); on the contrary, surface area of villi in cecum was significantly smaller in green season than that in dry season (p<0.05). However, no significant difference was observed in the rectum. The crypt depth in the duodenum, jejunum, and ileum was greater in green season than that in dry season (p<0.05), while there was no significant difference in cecum and rectum (p>0.05).

**Table-3:** The surface area (μm^2^) of villi and crypt depth (µm) in the intestine of yak in different grazing seasons.

Sections	Surface area of villi		p-value	Crept depth		p-value
	
	Dry season	Green season		Dry season	Green season	
Duodenum	28667.50±1497.43^B^	63970.821±3070.23^A^	0.005	60.94±3.36^B^	69.69±3.34^A^	0.361
Jejunum	175725.26±13610.75^B^	423924.33±21196.72^A^	0.016	45.42±2.67^B^	54.33±2.18^A^	0.012
Ileum	67296.19±3537.39^B^	99528.92±4812.31^A^	0.009	72.16±3.49^B^	85.86±3.57^A^	0.037
Cecum	72518.76±3115.58^A^	62789.833±3059.86^B^	0.006	69.28±3.01^A^	70.28±3.69^A^	0.907
Rectum	23586.24±1791.27^A^	222909.27±10631.81^A^	0.717	78.92±2.42^A^	76.056±3.26^A^	0.829

Means within rows with different superscript capital letters at same day are different (p<0.05)

### The submucosa and muscular thickness of the yak intestine in different grazing seasons

The submucosa and muscular thickness of intestine are summarized in Table-4. Submucosa thickness of duodenum, jejunum, ileum, and rectum was significantly higher in green season than that in dry season (p<0.05); on the contrary, the submucosa thickness of cecum was smaller in green season than that in dry season (p<0.05). The muscular thickness of jejunum, cecum, and rectum was greater in green season than that in dry season (p<0.05), while there was no significant difference between the muscular thickness in duodenum and ileum (p>0.05).

**Table-4:** The submucosa and muscular thickness (µm) of intestine of yak in different grazing seasons.

Sections	Submucosa thickness			Muscular thickness		
	
	Dry season	Green season	p-value	Dry season	Green season	p-value
Duodenum	54.35±2.79^B^	124.34±5.32^A^	0.037	425.154±20.67^A^	413.610±16.32^A^	0.589
Jejunum	79.87±3.88^B^	110.335±4.15^A^	0.025	140.078±7.95^B^	239.461±9.78^A^	0.024
Ileum	78.68±3.54^B^	109.815±4.36^A^	0.001	223.821±9.86^A^	218.880±10.59^A^	0.645
Cecum	186.49±9.65^A^	143.804±6.94^B^	0.009	269.436±12.52^B^	377.496±18.21^A^	0.004
Rectum	92.54±4.75^B^	153.878±7.37^A^	0.014	229.460±9.66^B^	324.705±12.94^A^	0.018

Means within rows with different superscript capital letters at same day are different (p<0.05)

## Discussion

The extent of morphophysiological variations in the digestive system of ruminants indicates a degree of adaptability for a particular feed [12]. The structure of the mucosal papillae in rumen was closely related with the animal feeding. When the nutritional quality of food is high, a large number of nutrients are degraded into short-chain fatty acids by microorganisms in rumen. Thus, the more plicae and mesh membranes can be formed, the more the surface area of papillae increases so that not only the surface area of original mucosa but also the absorption area in rumen increase [12,13]. These findings are in line with the results of the present study which showed that the values of the length and width of the rumen papillae were higher in the green season. Thus the surface and the absorption area were increased. On the contrary, during the dry season characterized by dry grass and lower temperature, the absorption surface decreased by shorter and narrower papillae. This seasonal pattern of the papillae morphology in the yak probably resulted from adaptations to extreme food and environmental conditions. This is consistent with Wang *et al*. [5] who reported that in the intensively reared group the length and width of rumen papillae were significantly increased when compared to the extensive group of cattle.

Changes in the development of enterocytes and the structure of villi and crypt are direct representation of intestinal environment and may be used as indicators of intestinal health [5]. Zitnan *et al*. [6] reported that the length of duodenal villi in the intensive group was significantly increased compared to the extensive group. Wang *et al*. [5] revealed that the height of villi increased in duodenum and jejunum of animals fed with concentrate diets. These coincide with our results about the height of villi in the duodenum and jejunum during the green season.

Furthermore, in the green season, we found that the crypt depth in the duodenum, jejunum, and ileum increased; these results were in agreement with that reported in the cattle intestine when fed with higher nutrients feedstuff [6].

Kreikemeier *et al*. [14] presumed the absorptive surface to represent the mucosal aspect of villi available for nutrient translocation, and the greatest absorptive surface is in the proximal area of the bovine small intestine and increases with increasing grain intake. These coincide with Zitnan *et al*. [6] and Wang *et al*. [5] finding results which revealed higher duodenal and jejunal villi surface area in animals fed increased nutrient concentrate diets. In the present experiment, same results are found that the villi surface area on duodenum, jejunum, and ileum increased in green season. These were not consistent with Liu *et al*. [15] reported that seasonal modulations are more distinct in the jejunum than in the duodenum and the ileum of the small intestines.

Although a little reference was reported that the submucosa thickness and muscular thickness of intestine changes due to diet, in this study, we found that the submucosa thickness and muscular thickness of intestine were increased with increased nutrient concentrate, it contributed to increase the absorption of nutrients in small intestine.

## Conclusion

In this research, we found that the seasonal changes result in the ruminal and intestinal morphology of yak, in particular, the length and width of papillae, villi, crypt, and submucosa. This fact was confirmed the functional advantages resulting from the ability to successfully adapt to a dry climate and diets, flat, open, and cold grassland may allow yak to overcome both water shortage and energy deficiency in winter.

## Authors’ Contributions

This work was carried out in collaboration between all authors. BAD, SQM, and ZRL: Designed the experimental procedures. BAD and SQM: Conducted the research work. XLL and SRM: Helped in data analysis and revision. All authors read and approved the final manuscript.
